# Working towards a better understanding of type 2 diabetes care organization with First Nations communities: a qualitative assessment

**DOI:** 10.1186/s13690-020-0391-8

**Published:** 2020-02-04

**Authors:** Lisa A. Wozniak, Jeffrey A. Johnson, Dean T. Eurich

**Affiliations:** 1 School of Public Health, University of Alberta, Edmonton, Alberta T6G 2E1 Canada; 2 Alliance for Canadian Health Outcomes Research in Diabetes, 2-040 Li Ka Shing Centre for Health Research Innovation, University of Alberta, Edmonton, Alberta T6G 2E1 Canada

**Keywords:** First Nations, Diabetes, Health services delivery, Qualitative description

## Abstract

**Background:**

Diabetes care is suboptimal in First Nations populations. Innovative and culturally-relevant approaches are needed to promote proactive organization of diabetes care for diabetes patients on-reserve in Canada. The Reorganizing the Approach to Diabetes care through the Application of Registries (**RADAR**) model is one strategy to improve care: an integrated disease registry and electronic health record for community healthcare workers with centralized care coordination. The aim of this study was to qualitatively assess the organization of type 2 diabetes care in participating communities in Alberta, Canada, at baseline prior to implementing RADAR.

**Methods:**

Using qualitative description, we purposefully sampled healthcare workers involved in diabetes care at each health center. We used the 5Rs framework (i.e., Recognize, Register, Resource, Relay, Recall) to inform the baseline assessment and conducted group interviews in 6 communities with 16 healthcare workers. Detailed notes were taken and validated by participants. Data was managed using ATLAS.ti 8 and analyzed using content analysis.

**Results:**

We found strong commitment and effort by local healthcare workers to support people living with type 2 diabetes in their communities. However, healthcare workers were limited in their ability to identify (i.e., recognize), track (i.e., register and relay) and manage (i.e., resource and recall) people with type 2 diabetes as proposed by the 5Rs framework. The organization of diabetes care was often reactive and dependent on patients’ abilities to navigate the health system. Interestingly, participants talked about the 5Rs in relationship to one another, not in a linear or isolated manner.

**Conclusions:**

Overall, the organization of diabetes care in participating communities did not align with the recommended approach of the 5Rs framework. In addition, we propose “reimagining” the 5Rs to reflect the interdependence and mediation of components situated within human and financial resources. This will better equip healthcare workers to assess, plan and execute organized and proactive diabetes care. However, the onus on people living with type 2 diabetes to engage with healthcare services remains a concern.

**Trial registration:**

ISRCTN.com, ISRCTN14359671.

## Background

Diabetes rates have reached staggering proportions among Indigenous peoples [1]. In Canada, the prevalence of diabetes is 3–5 times higher in First Nations than in the general population [2]. First Nations people experience a higher prevalence of diabetes-related complications and mortality [2]. Canadian data shows the mortality rate is 2–3 times higher for Aboriginal peoples than the general population with diabetes [3]. These concerns are exacerbated by suboptimal diabetes care, particularly in remote settings where many First Nations people live [2, 4–6]. Applying the Chronic Care Model [7] to diabetes care, with appropriate cultural context, could lessen the impact of diabetes among First Nations.

The Chronic Care Model promotes proactive management of diabetes at a population level through: team-based approach; self-management support of patients; decision support; clinical information systems; and resources and policies [8, 9]. When implemented in primary care settings, the model improves quality of care and outcomes [8, 10, 11]. Diabetes Canada guidelines recommend the 5Rs approach to organizing diabetes care within a Chronic Care Model framework: **R**ecognize (assess risk factors and screen); **R**egister (systematically track patients); **R**esource (support self-management through inter-professional teams); **R**elay (facilitate information sharing); and **R**ecall (timely review and reassessment) [8]. Few First Nations communities have the infrastructure, expertise, or resources to employ the 5Rs for diabetes care. Innovative and culturally-relevant approaches are needed to promote the proactive organization of diabetes care for people living with diabetes on-reserve in Canada [8, 12].

The Reorganizing the Approach to Diabetes care through the Application of Registries (**RADAR**) model was developed in collaboration with First Nations communities and OKAKI Health Intelligence Inc. (Calgary, Canada) (**OKAKI**), a private sector company with more than 10 years of experience working with First Nations communities in Alberta, Canada. We are evaluating the effectiveness of RADAR using a controlled trial study protocol reported elsewhere [13]. Briefly, the RADAR model uses centralized care coordinators (i.e., registered nurses) to work with local healthcare workers in participating First Nations communities to: (1) populate an electronic diabetes registry/electronic health record (**EHR**) (e.g., demographics, clinical, lab information) called the Community Assessment Response and Empowerment – CARE software platform (**CARE EHR**); and (2) assist, coordinate and monitor care at the population-level (e.g. identify care gaps, coordinate referrals) through the CARE EHR and telehealth approaches.

For this study, we aimed to qualitatively assess the organization of type 2 diabetes (**T2D**) care in participating First Nations communities at baseline *prior to implementing RADAR* as little is known about how T2D care is organized within the context of First Nations communities in Alberta. This information is vital to implementing quality improvement interventions like RADAR and to contextualize the findings for the larger controlled trial study. For example, we would not expect a change in outcomes because of RADAR if T2D care was organized in these communities using the 5Rs approach prior to implementing RADAR.

## Methods

### Study design

We used qualitative description [14, 15] to elicit healthcare workers’ perceptions of the current organization of T2D care in their communities. Qualitative description is appropriate when the goal is to produce a summary of a phenomenon, such as the organization of care. First Nations leaders and community health managers in participating communities reviewed and endorsed the project. Approval was obtained from the University of Alberta’s Health Research Ethics Board (Study ID Pro00048714). All participating healthcare workers provided verbal consent. We followed the consolidated criteria for reporting qualitative research (**COREQ**) in reporting this study [16]. More detail is provided in an additional file (Additional file 1).

### Setting

The implementation of RADAR is a collaborative effort with several First Nations communities from Treaty 6, 7 and 8 territories in Alberta, Canada. We restricted the qualitative baseline assessment to 6 communities to balance the demands of rigor with feasibility and timeliness. In addition, due to the staggered implementation of the controlled trial study, the baseline assessment would have taken several more years to complete if we included all of the participating communities. The 6 selected communities were diverse in context offering a range of perspectives regarding the current organization of T2D care. Health managers estimated that approximately 750 people with T2D reside in the 6 communities. The majority of care for people living in First Nations communities is delivered through federally funded, nurse-led homecare, community health, and diabetes programs [17]. Health services, including diabetes care, are primarily delivered by local, on-reserve, healthcare workers (e.g., registered nurses, licenced practical nurses, community health representatives) with limited access to primary care physicians and specialist services [18]. The 6 participating communities are situated approximately 30–180 min from a major urban center, where the majority of diabetes specialist services are located.

At baseline, all communities had Alberta Netcare (**Netcare**), a secure and confidential EHR that provides healthcare workers with patients’ health information including demographics, medications, laboratory test results, diagnostic images and reports, hospital discharge summaries, and surgeries. The health centres implemented the CARE EHR for their home care programs; however, it was not being used specifically for diabetes care.

### Data collection

With the assistance of health managers, we identified and purposefully sampled all healthcare workers involved in diabetes care at each health center. To achieve consensus regarding the organization of T2D care, respondents in each community were invited to participate in 1 group interview via telephone. A trained qualitative researcher (LAW) conducted the interviews using a semi-structured interview guide informed by the 5Rs framework (Additional file 2). Interviews lasted approximately 90 min. Detailed notes were taken and validated by participants for accuracy. All data were kept on a secure server or in locked files in a secure office with privileged access with only the research team having access to the data.

### Data analysis

We used content analysis [19] to examine the data [14]. LAW conducted the primary analyses using an integrated approach. First, the data was coded using the 5Rs framework. Then, we coded data not directly related to the framework. Last, we applied an inductive approach to identify emerging codes and concepts within, across, and outside of the 5Rs. We reviewed code definitions and emerging concepts at regular research team meetings, and discussed discrepancies to reach consensus. All data was managed with and queried using ATLAS.ti Version 8 (Berlin, Germany, Scientific Software Development GmbH) [20]. As described above, we drew on several strategies to ensure reliability, including methodological coherence, peer debriefing, and maintaining an audit trail [21, 22].

## Results

We conducted 6 group interviews, with a total of 16 individuals, between November 2014 and July 2017. Types of healthcare workers included: registered nurses (8); licenced practical nurses (3); health managers (2); and other (3) (i.e. community health representative, retinal photographer, registered dietician). Types of programs represented by participating healthcare workers included: administration (3); home care (6); community health (4); and diabetes program (3).

As demonstrated by their participation in RADAR, we found strong commitment and effort by local healthcare workers to support patients with T2D in their communities. However, healthcare workers reported being limited in their ability to identify, track and manage people with T2D. Below, we describe the organization of T2D care prior to implementing RADAR by the processes of identifying (i.e., Recognize), tracking (i.e., Register and Relay), and managing (i.e., Resource and Recall) of patients. We provide a summary the organization of T2D care at baseline by the 5Rs in Table 1.

**Table 1:** Summary of the organization of type 2 diabetes care by the 5Rs at baseline

RECOGNIZE: *Appropriately screen for & identify people with diabetes* ▪ Limited screening due to limited resources and patients screened elsewhere by other providers ▪ Used existing records/registries (e.g., chronic diseases lists, CARE EHR, Physician EMR) ▪ Relied on patient self-referral or engagement with healthcare system	
REGISTER: *Develop a means of tracking all patients with diabetes* ▪ Used patient charts or chronic diseases lists, paper-based and/or electronic ▪ Data entry in the context of limited resources was problematic ▪ Relied on patient self-referral or engagement with healthcare system	
RESOURCE: *Support self-management through inter-professional teams* ▪ Differential access to diabetes-related providers and services, including diabetes educators or programming ▪ Limited ability to support patients in context of limited resources ▪ Lack of collaborative care between healthcare providers ▪ Relied on patient self-referral or engagement with healthcare system	
RELAY: *Information sharing between patient & healthcare team members* ▪ Used Netcare, CARE EHR, or Physician EMR ▪ Facilitators: colocation of healthcare workers and providers; existing relationships ▪ Barriers: privacy concerns; limited access to data or data systems; or incompatibility of Physician EMRs with CARE EHR resulting in data entry	
RECALL: *Remind caregivers & patients of timely review & reassessment* ▪ Used CARE EHR, patient chart review, or chronic diseases lists; patient reminders ▪ Barriers: patients not engaging with healthcare system (e.g., no shows even when recalled); limited use of CARE EHR by all staff; time consuming, not knowing who had T2D)	

LEGEND: *EMR* Electronic Medical Record; *T2D* type 2 diabetes; *EHR* Electronic Health Record

### Identify T2D patients (Recognize)

Prior to implementing RADAR, healthcare workers reported limited screening to identify people with T2D. This was often because healthcare workers presumed that people were screened for T2D by physicians. When people were screened for and diagnosed with T2D by physicians, this information was not always relayed to the healthcare workers expected to perform subsequent diabetes care. When screening was conducted at the health centers, it relied on patient self-referral to health centres, including when patients were experiencing symptoms or attending special events (e.g., health fairs, diabetes walks). In addition, limited financial and human resources diminished healthcare workers’ ability to screen people who accessed their services.

Due to limited screening, healthcare workers primarily identified people with T2D through: patient self-referral after diagnosis by a physician; existing information sources or registries, including Netcare, department- and external program-based chronic diseases lists of patients (i.e., paper- and/or electronic-based), the CARE EHR, electronic medical records (**EMRs**), or databases (e.g., non-insured health benefits); and/or knowing the community or by word of mouth. Respondents recognized the problematic nature of only identifying those having T2D among people who accessed health services versus those who did not. Reported patient-level barriers to accessing health services included lack of transportation and/or fear, denial or apathy about diabetes.

### Track T2D patients (Register & Relay)

At baseline, healthcare workers primarily registered patients with T2D using patient charts, chronic diseases lists (paper- and/or electronic-based), the CARE EHR (narrative chart), and/or EMR. Of note, a diabetes registry was being built within an EMR in 1 community as part of a different quality improvement initiative. While the CARE EHR was available prior to implementing RADAR, its full capability was not used and limited to the narrative chart and client admission/service tracking, typically used only by home care staff.

Healthcare workers identified data entry as a barrier to registering patients with T2D, including entering paper-based data from records (e.g., patient charts and department- or program-based lists) to the CARE EHR. The burden of data entry was often situated in the context of limited financial and human resources, including inadequate staffing levels and/or competing priorities (e.g., immunizations, flu clinics) and limited compatibility of information systems (e.g. Physician EMRs with the CARE EHR). Despite this, the move from a paper-based to an electronic-based information system (i.e., the CARE EHR) indicated a positive transition from registering patients at an individual- to a panel-level. Regardless, the strategies used to register patients with T2D still relied on patient self-referral to healthcare services or were focused on already managed patients (e.g., home care patients), thus missing people who did not access these services.

At baseline, healthcare workers reported some degree of relaying clinical information between members of the healthcare teams (e.g., healthcare workers, physicians, specialists, pharmacists, patients); however, this was often done inconsistently. They reported using: information systems, including Netcare, the CARE EHR, or Physician EMRs; in-person communication when co-located; telecommunication (i.e., telephone, fax, or messaging); and/or established referral processes.

In contrast, there were examples of limited relaying of clinical information among healthcare teams. Not being co-located was reported as a barrier, with respondents reporting limited opportunities to share information with visiting physicians and specialists or staff housed in separate buildings. Respondents explained that a lack of awareness of and relationships between members of healthcare teams was a barrier, particularly to sharing clinical information with providers located outside of the communities that led to gaps in coordinated care. For example, despite being located in the same small community, healthcare workers at the health center and family physicians in a separate clinic had never formally met to discuss coordination of diabetes care until the OKAKI/research team brought them together to discuss implementing RADAR. During this face-to-face meeting, physicians learned that retinal teleophthalmology was available at their local health center. Until this point, physicians had sent patients to an urban center (~ 3 h travel) for a service available in the community. Additional barriers to relaying clinical information included: privacy issues, including the need for patient consent; patients accessing multiple points of care; and limited access to data (e.g., missing data) or information systems (e.g., only registered nurses had access to EMR or Netcare).

### Manage T2D patients (Resource & Recall)

Healthcare workers reported differential access to diabetes-related providers and programming by community. Respondents in all communities reported the availability of nurses (i.e., registered nurses and/or licenced practical nurses) and the following programs/ services: home care, foot care, and medical transportation. Most respondents reported some access to family physicians (e.g., rotating physicians visiting the health center 2 days/week), pharmacists, or dieticians and the following programs/services: laboratory services, diabetes programing, or retinal photography. It was less common for patients in the communities to have access to community health representatives, providers through primary care networks, certified diabetes educators, or specialists and the following programs/services: hospitals, medication review, or mental health services. We provide a detailed summary of the available providers and services in the communities as identified by respondents in Additional file 3.

In addition, respondents identified patient- and system-level barriers to the self-management of T2D. At the patient-level, they perceived limited patient engagement with healthcare services, including frequent “no shows” for appointments or tests. Rationales for patients’ limited engagement included: fear or denial of T2D; lack of transportation; or completing priorities, such as work. At the system-level, respondents reported limited financial (i.e., funding, equipment) and human (i.e., staffing, time, and expertise) resources as a barrier to healthcare workers supporting patients with self-management.

Respondents varied in their opinions regarding the appropriateness of care offered to support patients in self-management. They described efforts to provide appropriate care to patients by healthcare workers (e.g., offering holistic care, tailoring programs to cultural or community preferences, using preferred language of patients, or understanding food insecurity) or other providers (e.g., family physician was a community member or visiting specialists had experience working with Indigenous communities). Respondents in 1 community described educating other providers on “culturally sensitive” care. Regardless of these positive examples, respondents identified barriers to appropriate care including: lack of trust among some patients to share information about their culture, current context, or ideas about their care with providers; lack of understanding among providers of the context faced by patients (e.g., food insecurity or barriers to accessing care and managing their health), often leading to a judgemental attitude; or lack of traditional medicine offered in conjunction with other treatments.

Prior to implementing RADAR, respondents reported variable use of strategies for recall. Strategies used to remind healthcare workers to review and reassess client targets included: use of information systems, such as the CARE EHR or Physician EMR; patient chart review on a regular basis or prior to scheduled client appointments; and/or the use of manual chronic diseases lists of patients due for care specific to departments (e.g., foot care, retinal photography, or immunizations). While the CARE EHR was identified as a tool for recall, respondents identified barriers including use by home care staff but not by all healthcare workers. Thus, only some members of a patient’s care team captured information needed in the CARE EHR for healthcare workers to know who to recall and for what service. Lastly, a few respondents recognized that they used no systematic means of recall for healthcare workers.

Techniques used to recall patients consisted mainly of patient reminders for scheduled appointments either in-person or by telephone or mail. Respondents reported recalling patients to book overdue appointments or when lab values were outside of normal range; however, this was often limited to home care patients. Respondents in 1 community identified access to clinical information (e.g., lab values through Netcare) as a facilitator to recalling patients. Regardless, respondents identified several barriers to recalling patients. First, the onus was on patients to make appointments or “walk-in” to needed healthcare services, rather than being proactively recalled. This is troubling as respondents identified patient-level barriers to accessing care even when recalled, including: “no shows” or missed appointments often because they lacked transportation; no phone to receive reminder calls; and/or changing contact information. Additional barriers to recalling patients included the time-consuming nature of the activity (i.e., resources needed) or not knowing who had diabetes because patients sought diabetes care elsewhere.

## Discussion

Overall, we found that healthcare workers in the participating First Nations communities were limited in their ability to systematically identify, track, and manage patients with T2D *prior to implementing RADAR*. These challenges are not unique to the healthcare workers in these communities. Across settings and health care professions, T2D can remain undiagnosed for many years [23], indicating the lack of screening to identify asymptomatic individuals. Furthermore, our previous study describing the development and use of diabetes registries in primary care networks in Alberta revealed similar issues to those reported here, including: the use of existing registries to recognize patients with T2D rather than proactively screen; relying on patient self-referral thereby limiting reach to patients engaged with the healthcare system; and limited relay of, or access to, clinical information [24].

We found that care was often reactive and dependent on patients’ abilities to engage with and navigate the health system. This is particularly troubling given the patient-level barriers to accessing care. It is unclear whether limited patient engagement with healthcare services was a result of limited access to services or health behaviours, including the conscious or unconscious decision not to access services, or a combination of both. For example, historical trauma makes it likely that First Nations people avoid healthcare services [25]. Due to the inequitable diabetes care received by Indigenous peoples, including access to providers and programs, there have been calls for cultural mentorship to increase the knowledge of non-Indigenous providers of the social and historical context influencing patients healthcare decisions, and moving beyond cultural competency to understanding culture as a relational and fluid process [26].

**Fig. 1:**
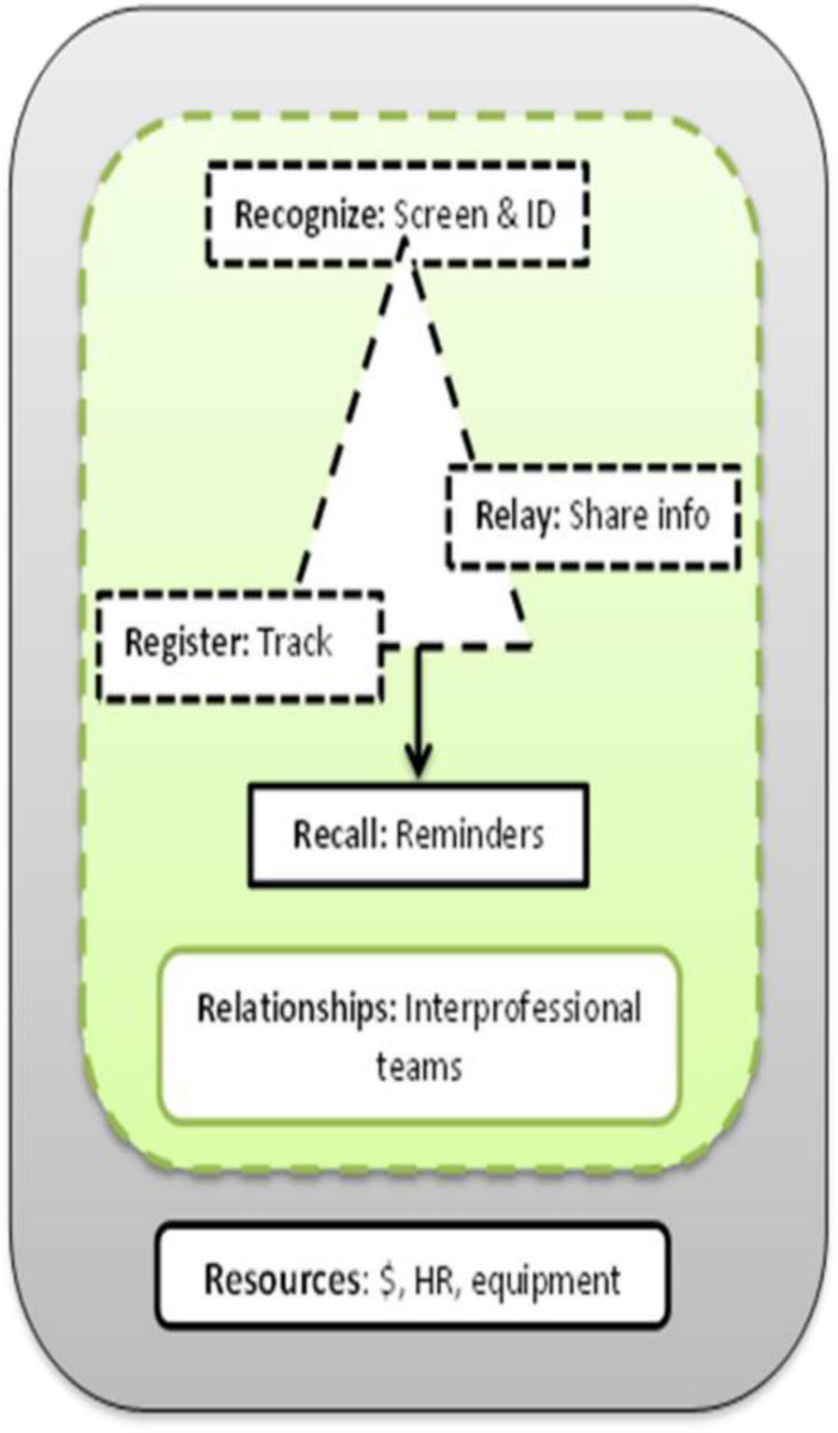
Reimagining the organization of diabetes care. ID: Identify. Info: Information. $: Financial resources. HR: Human resources

Lastly, we found that healthcare workers discussed the 5Rs of organizing care in relationship to one another, not in a linear or isolated manner. They situated the 5Rs in the context of relationships with other healthcare providers and the availability of sufficient financial and human resources. Thus, while the definitions of the 5Rs are distinct, they represent connected and overlapping processes situated in a broader context, particularly relationships and resources. Based on these results, we propose reimagining the 5Rs to 6Rs: replace the existing term “Resource” with “Relationships” and reflect the interdependence and mediation of components with dotted lines with all components situated within the 6th R of “Resources” (e.g., financial & HR) (Fig. 1).

The RADAR model has the potential to address most of the challenges to organizing diabetes care as reported in this qualitative study by operationalizing a 6Rs approach: Recognize, Register, Relay, Recall, Relationships, and Resources. In addition, RADAR can facilitate a community-based team approach to diabetes care that is culturally relevant and aligned with recommendations to improve diabetes care for First Nations peoples [1, 13]. This can be achieved by combining information systems and centralized care coordination to strengthen community capacity; it has been proposed that telehealth, while not a specific element of the Chronic Care Model, could facilitate components of the model [8]. For example, a centralized RN care coordinator, partnered with each community, can facilitate central registration (**Register**) of patients with T2D by populating the CARE EHR with specific diabetes measures, compiling information from multiple sources with the support of data entry clerks (**Resources**). They could support the relaying of clinical information (**Relay**) by supporting relationship-building between local healthcare workers and providers as well as addressing privacy concerns to ensure that those responsible for diabetes care have access to the information they need to support patients in self-management (**Relationships**). In addition, the RN care coordinators can support healthcare workers in recalling patients (**Recall**) who are not meeting targets or scheduled for regular appointments and coordinate care within (e.g., promote awareness of existing services) and outside of (e.g., specialty care) the community (**Relationships**). Indeed, centralized RN care coordinators represent essential human resources (**Resources**) for local healthcare workers, working to increase their capacity (i.e., knowledge and confidence) in delivering diabetes care to their patients.

While the onus remains on individuals to engage with the healthcare system, there are opportunities to systematically screen for T2D (**Recognize**) in this primary care setting as part of RADAR. This aligns with the clinical practice guidelines to screen more frequently in special populations with additional risk factors for diabetes [27]. Indeed, previous studies demonstrate that screening in rural or remote communities is possible when done appropriately (i.e., with respect, resources, and follow-up), including in Alberta [2, 28]. There is the potential for RADAR to expand, and be implemented by community outreach teams, taking the onus off of people to engage in traditional healthcare settings and potentially increasing the number of people recognized and offered coordinated diabetes care.

Regardless of access to centralized RN care coordinators, local healthcare workers are best positioned to provide appropriate direct patient care based on their existing relationships and understanding of the context of each community. For example, authors of a systematic review of telediabetes services found that the involvement of Indigenous health workers was an enabler, in part due to their role in communicating in local language and understanding of the community [29]. This approach is likely better than having RNs “parachute” in to the communities to provide direct patient care. This aligns with Diabetes Canada’s recommendation that the management of diabetes in Aboriginal peoples should follow the same clinical practice guidelines as those for the general population, with respect and sensitivity to language, cultural history, and traditional ways of knowing, using a holistic approach [2].

Our results should be interpreted in light of several limitations. Although we sampled a diversity of healthcare workers with knowledge of the organization of diabetes care in their health centres, our sample size was small. Also, while participating communities were diverse, our findings are based on the experiences of healthcare workers from 6 voluntary health centres that may not be representative of other health centres on-reserves. There is limited transferability of our results beyond the present context (i.e. sample and setting). Regardless, the strengths of this work include its qualitative descriptive approach and adding to the current literature by describing the organization of diabetes care in First Nations communities in Alberta. Further, this baseline assessment of T2D care *prior to implementing RADAR* will inform our overall evaluation of its effectiveness [13].

## Conclusions

Overall, we found that healthcare workers in the participating First Nations communities were limited in their ability to systematically identify, track, and manage patients with T2D. RADAR has the potential to address some of these issues. In addition, our reimagining of the 5Rs to 6Rs based on the results of this study may equip local healthcare workers to plan and execute organized diabetes care in their communities. It recognizes the interconnectedness of components and emphasizes relationships among healthcare providers to organize T2D care within the context of sufficient resources. Further, RADAR can support First Nations community capacity to organize T2D care. The model addresses many challenges identified at baseline through its electronic registry/EHR support and access to a centralized care coordinator. This model may be applied by organizations to improve the delivery of diabetes care. Regardless of the operationalization of a 6Rs approach through RADAR, the onus on T2D patients to engage with healthcare services remains a concern.

## Supplementary information

**Additional file 1:** Consolidated criteria for reporting qualitative studies (COREQ): 32-item checklist. We provide more detail on reporting this study using the consolidated criteria for reporting qualitative research (COREQ) checklist.

**Additional file 2:** Interview guide informed by the 5Rs framework. We provide the semi-structured interview guide informed by the 5Rs framework.

**Additional file 3:** Summary of available providers and services in communities identified by respondents. We provide a detailed summary the available providers and services in the communities as identified by respondents.

## Data Availability

The dataset supporting the conclusions of this article are not publicly available due to confidentiality but are available from the corresponding author on reasonable request.

## Abbreviations

CARE EHR: Community Assessment Response and Empowerment – CARE software platform
COREQ: consolidated criteria for reporting qualitative research
EHR: Electronic health record
EMR: Electronic medical record
Netcare: Alberta Netcare electronic health record
OKAKI: OKAKI Health Intelligence Inc. (Calgary, Canada)
RADAR: Reorganizing the approach to diabetes through the application of registries
T2D: Type 2 diabetes
