# Supplementary material for: Working towards a better understanding of type 2 diabetes care organization with First Nations communities: a qualitative assessment
Source: Arch Public Health. 2020 Feb 4;78:7. doi: 10.1186/s13690-020-0391-8 (PMC6998233; doi:10.1186/s13690-020-0391-8)
Supplement: Supplementary file 1 — Additional file 1: Consolidated criteria for reporting qualitative studies (COREQ): 32-item checklist. We provide more detail on reporting this study using the consolidated criteria for reporting qualitative research (COREQ) checklist. [file 13690_2020_391_MOESM1_ESM.doc]

**Supplemental Appendix: Consolidated criteria for reporting qualitative studies (COREQ): 32-item checklist**

Developed from: Tong A, Sainsbury P, Craig J. Consolidated criteria for reporting qualitative research (COREQ): a 32-item checklist for interviews and focus groups. *International Journal for Quality in Health Care*. 2007. Volume 19, Number 6: pp. 349 – 357.

| **No. Item** | **Guide questions/description** | **Response &/or page # reported on** |
| --- | --- | --- |
| **Domain 1: Research team and reﬂexivity** | | |
| *Personal Characteristics* | | |
| 1. Interviewer/facilitator | Which author/s conducted the inter view or focus group? | LAW conducted the group interviews; Page 6 |
| 2. Credentials | What were the researcher’s credentials? E.g. PhD, MD | MA; Unblinded title page |
| 3. Occupation | What was their occupation at the time of the study? | Research Associate |
| 4. Gender | Was the researcher male or female? | Female; Unblinded title page |
| 5. Experience and training | What experience or training did the researcher have? | LAW is a medical anthropologist with nearly 20 years of experience designing and conducting qualitative research studies and program evaluations. |
| *Relationship with participants* | | |
| 6. Relationship established | Was a relationship established prior to study commencement? | No. |
| 7. Participant knowledge of the interviewer | What did the participants know about the researcher? e.g. personal goals, reasons for doing the research | All participants were informed about the reasons for the research topic and the role and occupation of the interviewer as part of the research team. |
| 8. Interviewer characteristics | What characteristics were reported about the inter viewer/facilitator? e.g. Bias, assumptions, reasons and interests in the research topic | All participants were informed about the reasons for the research topic and the role and occupation of the interviewer as part of the research team. |
| **Domain 2: study design** | | |
| *Theoretical framework* | | |
| 9. Methodological orientation and Theory | What methodological orientation was stated to underpin the study? e.g. grounded theory, discourse analysis, ethnography, phenomenology, content analysis | Qualitative description as described by M. Sandelowski; Page 4 |
| *Participant selection* | | |
| 10. Sampling | How were participants selected? e.g. purposive, convenience, consecutive, snowball | Purposefully sampling; Page 6 |
| 11. Method of approach | How were participants approached? e.g. face-to-face, telephone, mail, email | With the help of community health managers, we identified all healthcare workers involved in diabetes care at each health center. All potential participants were invited to participate in the group interview through email. |
| 12. Sample size | How many participants were in the study? | A total of 16 participants (out of a possible 20 who were invited) were included in the assessment; Page 7 |
| 13. Non-participation | How many people refused to participate or dropped out? Reasons? | 4 individuals who were invited to participate in the assessment did not response to the email invitation. |
| *Setting* | | |
| 14. Setting of data collection | Where was the data collected? e.g. home, clinic, workplace | Group interviews were conducted by telephone. The interviewer was located in a private room at the central office of the research team. The participants were located in a private room at their health center. |
| 15. Presence of non-participants | Was anyone else present besides the participants and researchers? | The interviewer was the only person present at her location. It is unclear whether people other than the participants were present in the private room at their health centers as the interviewer could not see the participants. |
| 16. Description of sample | What are the important characteristics of the sample? e.g. demographic data, date | Types of healthcare workers and programs represented are described; Page 7 |
| *Data collection* | | |
| 17. Interview guide | Were questions, prompts, guides provided by the authors? Was it pilot tested? | The semi-structured interview guide is presented in Table 1; Page 6 |
| 18. Repeat interviews | Were repeat inter views carried out? If yes, how many? | No, inferred on page 6 |
| 19. Audio/visual recording | Did the research use audio or visual recording to collect the data? | Detailed notes were taken and validated by participants for accuracy; Page 6 |
| 20. Field notes | Were ﬁeld notes made during and/or after the interview or focus group? | Detailed notes were taken and validated by participants for accuracy; Page 6 |
| 21. Duration | What was the duration of the inter views or focus group? | Interviews lasted approximately 90 minutes; Page 6 |
| 22. Data saturation | Was data saturation discussed? | Data saturation was not relevant in this assessment as we identified and invited *all* healthcare workers involved in diabetes care at each health center. As such, there were no other people to invite to participant in the assessment who possessed expert knowledge of the organization of diabetes care at the 6 participating health centers; inferred on page 6. |
| 23. Transcripts returned | Were transcripts returned to participants for comment and/or correction? | Detailed notes were taken and validated by participants for accuracy; Page 6 |
| **Domain 3: analysis and ﬁndings** | | |
| *Data analysis* | | |
| 24. Number of data coders | How many data coders coded the data? | LAW conducted the primary analyses using an integrated approach…. We reviewed code definitions and emerging concepts at regular research team meetings and discussed discrepancies to reach consensus; Page 6 |
| 25. Description of the coding tree | Did authors provide a description of the coding tree? | LAW conducted the primary analyses using an integrated approach. First, the data was coded using the 5Rs framework. Second, we coded data not directly related to the framework. Lastly, we applied an inductive approach to identify emerging codes and concepts within, across, and outside of the 5Rs; Page 6 |
| 26. Derivation of themes | Were themes identiﬁed in advance or derived from the data? | LAW conducted the primary analyses using an integrated approach. First, the data was coded using the 5Rs framework. Second, we coded data not directly related to the framework. Lastly, we applied an inductive approach to identify emerging codes and concepts within, across, and outside of the 5Rs; Page 6 |
| 27. Software | What software, if applicable, was used to manage the data? | All data was managed with and queried using ATLAS.ti Version 8; Page 8 |
| 28. Participant checking | Did participants provide feedback on the ﬁndings? | As the baseline assessment unfolded, community participants reviewed and provided feedback on the findings. |
| *Reporting* | | |
| 29. Quotations presented | Were participant quotations presented to illustrate the themes/ﬁndings? Was each quotation identiﬁed? e.g. participant number | No transcripts were produced as detailed notes were taken during group interviews, rather than using audio recording. As such, direct quotes are not provided. However, the detailed notes were validated by participants for accuracy, which is reflected in the results section; Pages 7-13 |
| 30. Data and ﬁndings consistent | Was there consistency between the data presented and the ﬁndings? | Yes; Pages 7-14 |
| 31. Clarity of major themes | Were major themes clearly presented in the ﬁndings? | Yes; Pages 7-14 |
| 32. Clarity of minor themes | Is there a description of diverse cases or discussion of minor themes? | Discussion of major and minor findings; Pages 7-14 |
