# Supplementary material for: Working towards a better understanding of type 2 diabetes care organization with First Nations communities: a qualitative assessment
Source: Arch Public Health. 2020 Feb 4;78:7. doi: 10.1186/s13690-020-0391-8 (PMC6998233; doi:10.1186/s13690-020-0391-8)
Supplement: Supplementary file 2 — Additional file 2: Interview guide informed by the 5Rs framework. We provide the semi-structured interview guide informed by the 5Rs framework. [file 13690_2020_391_MOESM2_ESM.doc]

**Supplemental Appendix: Interview guide informed by the 5Rs framework**

| **RECOGNIZE**: |
| --- |
| 1. Describe your current practices of identifying people with T2D in your community. In other words, how do you know who has T2D in your community? |
| 1. Describe your centre’s practice or protocol for screening for T2D. |
| 1. What are the 3 main barriers to identifying who has T2D in your community? |
| 1. What are the 3 key facilitators to identifying who has T2D in your community? |
| **REGISTER:** |
| 1. Describe how you record, organize, and track information for people with T2D in your community. |
| 1. What are the 3 main barriers to recording, organizing and tracking information for people with T2D? |
| 1. What are the 3 key facilitators to recording, organizing and tracking information for people with T2D? |
| **RESOURCE:** |
| 1. What are the current resources for people with T2D in your centre and/or in the community (e.g., family physicians, diabetes educators, pharmacists, dieticians)? |
| 1. In your opinion, are these resources (culturally) appropriate? |
| 1. For what visits/services must people travel? |
| 1. What isn’t working around resourcing care for people with T2D in your community? |
| 1. What is working around resourcing care for people with T2D in your community? |
| **RELAY:** |
| 1. How do you/staff share clinical information with a person with T2D and other healthcare providers that people may see about T2D? Provide examples. |
| 1. What are the 3 main barriers to sharing clinical information? |
| 1. What are the 3 key facilitators to sharing clinical information? |
| **RECALL:** |
| 1. How do people with T2D usually access the services at your centre? |
| 1. How do you remind healthcare providers in your clinic to review and assess targets for people with T2D? |
| 1. How do you remind healthcare providers to recall people with T2D for regular visits? |
| 1. How do you remind people with T2D of upcoming appointments, follow-up, or needed tests? |
| 1. What are the 3 main barriers to recalling people with T2D? |
| 1. What are the 3 key facilitators to recalling people with T2D? |
| Is there anything else you’d like to share with me that we haven’t covered? |

**LEGEND:**

T2D: type 2 diabetes
