# Supplementary material for: Working towards a better understanding of type 2 diabetes care organization with First Nations communities: a qualitative assessment
Source: Arch Public Health. 2020 Feb 4;78:7. doi: 10.1186/s13690-020-0391-8 (PMC6998233; doi:10.1186/s13690-020-0391-8)
Supplement: Supplementary file 3 — Additional file 3: Summary of available providers and services in communities identified by respondents. We provide a detailed summary the available providers and services in the communities as identified by respondents. [file 13690_2020_391_MOESM3_ESM.doc]

**Supplemental Appendix: Summary of available providers and services in communities identified by respondents**

| **Community #:** | **1** | **2** | **3** | **4** | **5** | **6** | **Total: Yes or Limited** |
| --- | --- | --- | --- | --- | --- | --- | --- |
| **Healthcare providers/workers** | | | | | | | |
| Nurses (Registered and/or Licensed Practical Nurses) | Yes | Yes | Yes | Yes | Yes | Yes | 6 |
| Family Physicians | Yes | Limited | Limited | Yes | Yes | Yes | 6 |
| Pharmacists | Yes | No | Yes | Yes | Yes | Yes | 5 |
| Dietician | Yes | No | No | Yes | Limited | Yes | 4 |
| Community Health Representative | NR | NR | NR | Yes | NR | Yes | 2 |
| Primary Care Network | No | No | Yes | NR | NR | Yes | 2 |
| Certified Diabetes Educator | NR | NR | NR | NR | No | Yes | 1 |
| Specialists | No | No | No | No | No | Limited | 1 |
| Occupational Therapist/ Physical Therapist/ Respiratory Therapist | NR | NR | NR | Yes | NR | NR | 1 |
| **Services/Programs** | | | | | | | |
| Foot Care | Yes | Yes | Yes | Yes | Yes | Yes | 6 |
| Home Care | Yes | Yes | Yes | Yes | Yes | Yes | 6 |
| Medical Transportation | Yes | Yes | Yes | Yes | Yes | Yes | 6 |
| Laboratory services | Yes | No | No | Yes | Yes | Yes | 4 |
| Diabetes programming | No | No | Yes | Yes | No | Yes | 3 |
| Retinal photography | Yes | No | No | Yes | NR | Yes | 3 |
| Hospitals | Yes | No | Yes | No | No | No | 2 |
| Medication review | Yes | NR | NR | No | Yes | NR | 2 |
| Mental health | Yes | NR | NR | Yes | NR | NR | 2 |
| Wound care | NR | NR | No | Yes | NR | NR | 1 |

**LEGEND:**

Yes: Available in community

No: Not available in community

Limited: Limited access / availability in community

NR: Not reported; no access / availability in community
